# Aligned or misaligned: Are public funding models for speech-language pathology reflecting recommended evidence? An exploratory survey of Australian speech-language pathologists

**DOI:** 10.1016/j.hpopen.2024.100117

**Published:** 2024-03-07

**Authors:** T. Nickless, B. Davidson, S. Finch, L. Gold, R. Dowell

**Affiliations:** a Department of Audiology & Speech Pathology, The University of Melbourne, Australia; b Word By Mouth Speech Pathology, Melbourne, Australia; c Statistical Consulting Centre, The University of Melbourne, Australia; d Deakin Health Economics, School of Health & Social Development, Deakin University, Australia; e The Royal Victorian Eye & Ear Hospital, Melbourne, Australia

**Keywords:** Quantitative research, Public funding models, Speech-language pathology

## Abstract

Government subsidised funding arrangements serve as an essential medium for families to access private speech-language pathology (SLP) services in Australia. This study aimed to investigate whether, from a provider perspective, contemporary public funding models (PFMs) align with best-available scientific evidence for management of children and young persons with swallowing and communication disorders within Australian private-practice settings. This exploratory study was distributed to paediatric speech-language pathologists throughout Australia via an online survey. A total of 121 valid surveys were completed by Australian speech-language pathologists with divergent career experiences. In comparing three familiar PFMs using mixed effects logistic regression models to estimate odds ratios, results indicated that perceived congruence with recommended scientific evidence for SLP management varied across PFMs: the odds of failing to align with scientific evidence was 4.92 times higher for Medicare’s Chronic Disease Management Plan (MBS_CDMP) than for the National Disability Insurance Scheme; and 7.40 times higher in comparison to Medicare’s Helping Children with Autism initiative. This study is the first to report on (in)congruence between PFMs that provide access to independent Australian SLP services for children and young persons and best available scientific evidence to inform clinical practice. Participants identified that: (a) four out of seven contemporary PFMs were unfamiliar to speech-language pathologists; and (b) MBS_CDMP initiative failed to align with the evidence-base for best scientific SLP management.

## Highlights

• Public funding models assist families in accessing critical primary care allied health services such as speech-language pathology.
• Policy architects designing healthcare funding must ensure that public funding models are congruent with best-available scientific evidence.
• More research is needed on public funding models within private-practice settings and their role in boarder healthcare systems.

## 1 Introduction

The diversity of contemporary public funding models (PFMs) available for children and young persons [1] with communication and swallowing disorders enables access to speech-language pathology (SLP) services through Australian private-practice providers. In Australia, SLP management is supported by a medley of funding typologies (i.e., health insurance [e.g., Medicare]; social insurance [e.g., National Disability Insurance Scheme]; third party insurance [e.g., transport accident insurance]; and education [public, independent and Catholic systems]; [2] which offer various funding streams via PFMs for private allied health services such as SLP. Due to the historical nature of how these PFMs were established [3], [4], some PFMs (e.g., Medicare’s Chronic Disease Management Plan [MBS_CDMP]) have not kept pace with the escalating cost in accessing SLP services [5]. The variation in monetary support of PFMs from the public purse has fostered a culture where out-of-pocket expenses are borne by consumers. The premise of universal healthcare in Australia supported by PFMs has, therefore, been undermined and threatened by increasing out-of-pocket costs in accessing crucial, primary care services. Such circumstances have subsequently impacted on government health-economic objectives of equity, efficiency and acceptability [2], [6], Table III, p.467). In addition, a body of international evidence exists where concerns have been raised over: (a) dosage principles that produce ineffective SLP management due to limited therapeutic inputs [7], [8], [9]; and (b) identification of which individuals should receive intervention [10], [11].

### 1.1 Growing demand for private-practice speech-language pathology services

Speech Pathology Australia, the national peak body representing over 14,000 Certified Practising Speech Pathologists in Australia [12] or approximately 70 percent of Australian speech-language pathologists [13], developed a unique algorithm using data from the Australian Bureau of Statistics to identify the future level of need for SLP services based on population-level attributes of socioeconomic disadvantage [14]. Increased population demand for SLP services identified by Speech Pathology Australia’s algorithm correspondingly impacts on future demand and access to PFMs. Thus, demand for public funding to access SLP services via private-practice providers will conjointly increase.

### 1.2 Dose, frequency and intensity: Instruments used in speech-language pathology management

Speech-language pathologists are guided by concepts of dose, frequency and intensity (also known as differential treatment intensity [DTI]) to deliver optimal healthcare [15], [16]. The evidence surrounding differential treatment intensity is emerging as a vital measurement and outcome instrument within specific treatment areas of SLP. Yet, anecdotal accounts from both individuals with communication and swallowing disorders and speech-language pathologists providing critical and timely services are consistently reporting failures of PFMs to reflect evidence-based practice (EBP). The efficacy of interventions through reporting of differential treatment intensity in the literature (see Table 1) has provided the SLP profession with guidance to replicate best scientific evidence outcomes. Even with repeated reporting in the literature of gold standard SLP management through principles of differential treatment intensity, a crucial link is missing between the evidence of differential treatment intensity research and the alignment of PFMs in reflecting evidence-based practice to achieve optimal outcomes for individuals with communication and swallowing disorders.

**Table 1:** Examples of mapping recommended evidence based practice for SLP management against selected Australian PFMs.

*Clinical SLP Practice Area*	*Study*	*Evidence Type*	*Differential Treatment Intensity (DTI) recommendations*	*n*	*Total number of sessions: to achieve most effective EBP*	*Mapping to familiar PFM*					
						*BL_1_ = NDIS ^a^*		*BL_2_ = MBS_CDMP ^b^*		*BL_3_ = MBS_HCWA ^c^*	
						*Does SLP management meet PFM criteria?*	*Does PFM align with EBP?*	*Does SLP management meet PFM criteria?*	*Does PFM align with EBP?*	*Does SLP management meet PFM criteria?*	*Does PFM align with EBP?*
*Fluency*	[17]	*RCT*	*Study compared individual* versus *group intervention. To reach Stage 2 of intervention, up to 14.3 h for individual and 9.2 h for group intervention. ^d^*	*54*	*up to 19*	***✓***	***✓***	***✓***	***✘***	***✓***	***✓***
*Speech – phonology*	[18]	*Three case studies: single case experimental designs*	*2 x up to 30 min sessions per week with a minimum dose of more than 50 trials.*	*Study 1 = 14* *Study 2 = 4* *Study 3 = 4*	*up to 42*	***✓***	***✓***	***✓***	***✘***	***✓***	***✘***
*Speech – phonology*	[19]	*RCT*	*3 sessions per week for a total of 8 weeks using a multiple opposition intervention.*	*54*	*24*	*✓*	*✓*	***✓***	***✘***	***✓***	***✘***
*Speech – childhood apraxia of speech*	[20]	*RCT*	*12 x 1-hour intensive sessions, scheduled 4 days per week, over 3 weeks ^d^. Dose was controlled across two intervention programs, ReST ^e^ and NDP3.^f^*	*26*	*12*	***✓***	***✓***	***✓***	***✘***	***✓***	***✓***
*Language – phonological awareness*	[21]	*Non-RCT*	*2 x 60 min sessions per week for a total of 20 sessions up to 4.5 months.*	*91*	*20*	***✓***	***✓***	***✓***	***✘***	***✓***	***✓***
*Language – past tense*	[22]	*Retrospective, single case experimental design*	*2x 30 min sessions per week is more effective than once weekly intervention for 10 weeks.*	*29*	*20*	***✓***	***✓***	***✓***	***✘***	***✓***	***✓***
*Language – morphosyntax*	[23]	*Single subject multiple-baseline design*	*1 x 30 min session per week for 20 weeks (10 h).*	*8*	*20*	***✓***	***✓***	***✓***	***✘***	***✓***	***✓***
*Language - stimulation*	[24]	*Large-scale effectiveness case series trial*	*The program consisted of parent group sessions (maximum 12 parent participants only) and individual coaching sessions (with child) using ITTTT ^g^. Individual coaching sessions were scheduled approximate 2*–*3 weeks between group sessions.*	*273*	*up to 9*	*✓*	*✓*	***✘***	***✘***	***✓***	***✓***
*Literacy – basic reading & reading comprehension*	[25]	*RCT*	*Daily intervention x 45-minute sessions up to 26 weeks (approximately 76.5 h).*	*72*	*up to 130*	***✓***	***✓***	***✓***	***✘***	***✓***	***✘***
*Literacy –* *early literacy development*	[26]	*Single subject uncontrolled design*	*1 x 15 min session per week over 20 weeks.*	*23*	*20*	***✓***	***✓***	***✓***	***✘***	***✓***	***✓***
*Swallowing- feeding*	[27]	*RCT, prospective design*	*Two study groups: (i) 10 x 30*–*60 min weekly sessions; or (ii) 10 x 30*–––*60 min in one week -intensive.*	*68*	*10*	***✓***	***✓***	***✓***	***✘***	***✓***	***✓***
*Swallowing - feeding*	[28]	*Retrospective cohort controlled design*	*5 x daily sessions per week of feeding therapy with a SLP.* *3 x sessions per week with OT, including one co-treat with SLP, over 5 weeks.*	*23*	*30*	***✓***	***✓***	***✓***	***✘***	***✓***	***✘***
*Voice*	[29]	*Systematic Review*	*The results show that voice therapy lasts an average of 9.25 weeks distributed over 10.87 sessions of mostly 30 (36.36 %) or 60 min (27.27 %) and occurs once (34.55 %) or twice (28.18 %) per week. Total face-to-face time with the patient is average 8.17 h.*	*47*	*up to 11*	***✓***	***✓***	***✓***	***✘***	***✘***	***✓***
*Voice –* *nodules*	[30]	*RCT*	*1 x weekly session for indirect or direct voice therapy for 8 to 12 weeks.*	*114*	*up to 12*	***✓***	***✓***	***✓***	***✘***	***✘***	***✓***

*Note:****✘****denotes ‘No’;****✓****denotes ‘Yes’; RCT = randomised contolled trial; ^a^ NDIS = National Disability Insurance Scheme; as detailed in sections 24 and 25 (*[31]*, 2013, ss 24 and 25); ^b^ MBS_CDMP = Medicare Chronic Disease Management Plan; as of 1st March 2023, both Helping Children with Autism (HCWA) and Better Start for Children with Disability (BS) have been categorised under a new Medicare Benefit Scheme (MBS) item titled Complex Neurodevelopmental Disorders*[32]*,^c^ MBS_HCWA = Medicare Helping Children with Autism; ^d^ intervention was completed in group formats; intervention can be replicated in 1:1, individualised treatment programs; ^e^ ReST = Rapid Syllable Transition Treatment ^f^ NDP3 = Nuffield Dyspraxia Programme – Third Edition; ^g^ ITTTT = It Takes Two To Talk..*

### 1.3 Which public funding models are incongruent with scientific evidence? A case of mapping of differential treatment intensity with public funding models

The Australian SLP profession is committed to contributing to the emerging evidence base and translating this scientific evidence into clinical practice. Speech-language pathologists aim to implement evidence-based practice and uphold tenets of trust (e.g., making clinically appropriate healthcare decisions; maximising funding provisions to produce best outcomes through application of best scientific evidence to SLP management; and preserving ethical values and principles when managing funding provisions [2], [33], [34], [35]. Speech-language pathologists face barriers to achieving this aim where contemporary PFMs do not align with best scientific evidence. Table 1 presents PFM eligibility as per corresponding legislation, guidelines and criteria together with an examination of scientific evidence to determine (in)congruence with contemporary PFMs (Medicare Chronic Disease Management Plan [36], [37]; Medicare Helping Children with Autism [36], [38]; National Disability Insurance Scheme [31], ss 24, 25, 33[1][b], 34[1]]; National Disability Insurance [39]]).

Our previous qualitative studies [2], [33] investigated the experiences and perceptions of twenty Australian speech-language pathologists with knowledge of access to public funding by children and young persons with communication and swallowing disorders for SLP management through private-practice operators [2]. It is not known whether these views are representative of the broader population of SLP providers. By mapping scientific differential treatment intensity findings for particular communication and swallowing disorders with contemporary PFMs (see Table 1), improved efficiencies including cost effectiveness and time efficiencies can be identified (e.g., attaining person-centred healthcare goals over shorter timeframes; and ensuring early intervention SLP management for maximum benefit).

This exploratory, descriptive study aimed to investigate speech-pathologists’ perspectives on congruence between best-available scientific evidence for SLP management and contemporary PFMs for children and young persons with communication and swallowing disorders within Australian private SLP practices. Therefore, the objectives of this study were:

(a) to investigate whether contemporary PFMs accessed by children and young persons (i.e., the population) through Australian private-practice SLP providers are perceived as congruent with contemporary scientific evidence for SLP management;
(b) to determine the familiarity of contemporary PFMs amongst Australian private-practice SLP providers;
(c) to rate contemporary PFMs used by Australian private-practice speech-language pathologists against best-available scientific evidence for SLP management; and
(d) to identify and rank contemporary PFMs by Australian private-practice speech-language pathologists in achieving client goals.

## 2 Materials and methods

### 2.1 Research design

Speech-language pathologists from all Australian states and territories were recruited to participate in an online survey (see Supplementary Material I: Survey) via: (a) a series of repeated advertisements on social media (e.g., Twitter); and (b) an email flyer sent to special interest groups of private-practice practitioners delivering services throughout Australia affiliated with Speech Pathology Australia, the peak national body representing Australian speech-language pathologists (i.e., Victorian Independent Practitioners Network; Queensland Private Practice Network; Private Speech Pathologists Association of Western Australia etc.).

### 2.2 Survey design & sampling

Given that the total population of Certified Practising Speech Pathologists practising in private-practice settings within Australia was estimated as n = 5819 [40] at the time of data collection, an estimated sample size of n = 361 was proposed to attain a margin of error of 5 % on a 95 % confidence interval for a proportion. This was quantified using a sample-size calculator [41] and supported through reference to other exploratory studies with similar precision of margin of error on estimates [42], [43].

Participants were required to satisfy the following inclusion criteria: (a) held financial membership of Speech Pathology Australia; (b) maintained status of Certified Practising Speech Pathologist, a legislative requirement for the provision of service through many PFMs; (c) were currently practising in private-practice settings; and (d) had experience practising within a paediatric caseload in any Australian state or mainland territory. If participants were unable to validate the above inclusionary criteria (see Supplementary Material I: Survey, questions 1–3), they were unable to advance to subsequent questions of the survey. After providing consent, participants answered a series of multiple choice, rank order, rating scale and short answer questions (see Supplementary Material I: Survey).

### 2.3 Data collection & analysis

Data was collected via an online survey between January and March 2022 and then analysed according to our study’s research objectives. To validate our previous findings of reported incongruence between contemporary funding models and the latest scientific evidence by speech-language pathologists [2], [33], a process of methodological and data triangulation was used to minimise any potential measurement, sampling or procedural bias [44]. Specific survey questions and permissible responses that correlate with our investigation are presented in Supplementary Material II: Specific survey questions (for complete survey, see Supplementary Material I: Survey). From the original survey choices of (1) *does not align*, (2) *aligns;* (3) *neither aligns nor does not align*; and (4) *not familiar*, response data were recoded to create binary variables to determine (in)congruence: (0) *other* (i.e., survey choices of *aligns; neither aligns nor does not align;* and *not familiar);* and (1) *does not align*. This was undertaken as a technique to simplify the analysis by converting categorical variables on alignment to binary variables to assist with interpretation of results.

Data were then analysed using a range of statistical methods. A standard method for analysis of binary outcomes is logistic regression; however, we chose an extension of logistic regression as there were repeated measurements from the same individual participants. Mixed effects logistics regression (MELR) was used to compare *does not align* (i.e., misaligned or incongruent with evidence-based practice) responses according to PFMs. Mixed effects logistics regression allowed for random effects of individual participants across repeated measures (i.e., survey questions). Importantly, mixed effects logistics regression permitted formal statistical comparisons between specific PFMs. Analysis was carried out employing Genstat Version 18 [45] and figures were generated using Minitab Version 20 [46].

To determine the strength of association between two binary variables (e.g., in our case comparing a baseline (BL) such as *familiar* PFMs [i.e., a PFM used to access private-practice SLP services by the population that are known to the research participant] with that of other *familiar* PFMs), odds ratios were employed. This is the ratio of the odds of an outcome occurring in the baseline PFM to the odds of the same outcome occurring in other selected contemporary PFMs. As a statistical measure, odds ratios can, in principle, take any positive value where values: (a) >1 indicate that the odds are higher in the other *familiar* PFM than in the baseline; (b) <1 indicate that the odds are lower in the other *familiar* PFM than in the baseline; and (c) equal to 1 indicates equal odds for both PFMs [47].

### 2.4 Selection of public funding model

Our survey investigated seven contemporary PFMs (see Supplementary Material III: Part A: Contemporary public funding models). We distinguished between PFMs that were *familiar* and those PFMs that were *unfamiliar* to participants. To determine familiarity of PFM, we removed data where participants selected the survey variable, *not familiar* (also see section below, *3.2 Familiarity of Public Funding Model*)*.* We assumed that the three remaining variables (i.e., [1] *does not align*; [2] *aligns; and* [3] *neither aligns nor does not align*) implied familiarity of PFMs. To be included in mixed effects logistics regression, a PFM had to be familiar to 60 % or more of respondents (also see below, *3.2 Familiarity of Public Funding Model*).

All SLP treatment areas identified in Table 1 met PFM criteria (i.e., NDIS - reasonable and necessary supports; social and economic participation [31], ss 33[1][b], 34 [1]]; MBS_CDMP - chronic condition and complex care [37], Medicare Benefits Schedule Item 10970]; MBS_HCWA – early intervention services for children undergoing diagnosis/assessment or treatment for autism [48], Medicare Benefits Schedule Items 82005, 82020]). Additionally, SLP practice areas highlighted in Table 1 were selected based on suitability of provision of service through private-practices (i.e., settings where Response-to-Intervention models can be delivered, for example Tier 3 [one:one] and Tier 2 [small group]; [49]).

Approval for this research was provided by The University of Melbourne, Faculty of Medicine, Dentistry & Health Sciences, Office of Research Ethics and Integrity (*reference: 2021*-*23153-24445*-*3*) prior to commencement of the study.

## 3 Results

### 3.1 Participants

One hundred and twenty-one speech-language pathologists participated in this study, with 94.2 % female reflecting current gender workforce patterns [14]. Participants’ ages ranged from 20 to 69 years, with a combination of early career (i.e., 0–5 years; 32.2 %), mid-career (i.e., 6–15 years; 31.4 %) and experienced (i.e., 16+ years; 36.4 %) speech-language pathologists. Participants reported a diversity of employment arrangements (see Supplementary Material IV: Participant characteristics), with the most common groups being clinician (38.8 %) and owner of practice & clinician (42.2 %).

### 3.2 Familiarity of public funding model

To determine familiarity of PFM, we calculated percentages for each of the seven contemporary PFMs investigated (see Supplementary Material V: Participant responses for public funding models reported as “*familiar*”). We then considered three conditions referred to as *alignment variables* (i.e., [1] *does not align*; [2] *aligns*; and [3] *neither aligns nor does not align*) for each individual PFM (see Table 2).

**Table 2:** Alignment variables for individual public funding models reported as “*familiar*” by participants (percent) ^a.^

Alignment variable	Total for all PFMs	NDIS ^b^*n = 121*	MBS_CDMP ^c^*n = 112*	MBS_HCWA ^d^ n = 85	MBS_BS ^e^*n = 64*	MBS_AHS ^f^*n = 44*	IS ^g^*n = 64*	TP ^h^*n = 24*
[1] does not align	46.7	33.1	67.9	42.4	37.5	63.6	50.0	33.3
[2] aligns	32.9	41.3	16.1	37.6	39.1	25.0	31.3	37.5
[3] neither aligns nor does not align	20.4	25.6	16.1	20.0	23.4	11.4	18.8	29.2

Notes: ^a^ For the purpose of decimal rounding, cumulative percentages of PFMs may not equal 100; ^b^ NDIS = National Disability Insurance Scheme; ^c^ MBS_CDMP = Medicare Benefit Schedule – Chronic Disease Management Plan; ^d^ MBS_HCWA = Medicare Benefit Schedule – Helping Children with Autism; ^e^ MBS_BS = Medicare Benefit Schedule – Better Start; ^f^ MBS_AHS = Medicare Benefit Schedule – Allied Health Services for Aboriginal Torres Strait Islander Decent with Health Checks; ^g^ IS = Independent Schools; ^h^ TP = Third Party.

**Fig. 1:**
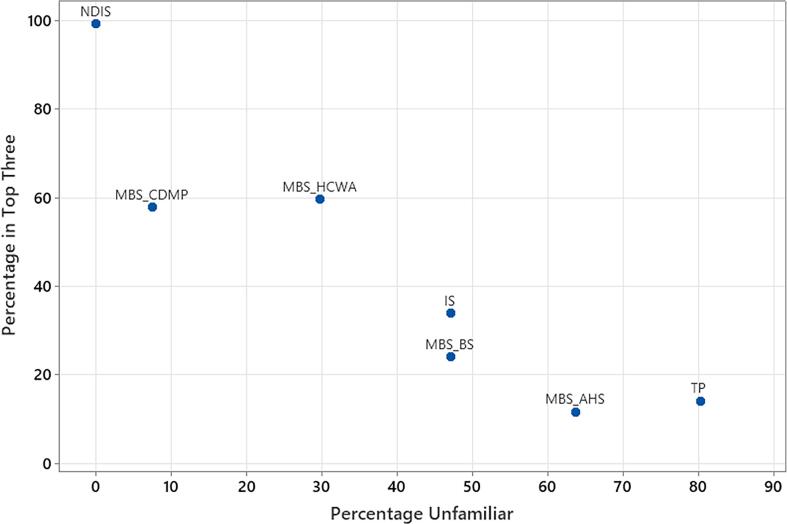
**Percentage of PFMs in Top Three in achieving SLP goals versus Percentage of PFMs unfamiliar to participants.***Note: (a) NDIS = National Disability Insurance Scheme; (b) MBS_CDMP = Medicare Chronic Disease Management Plan; (c) MBS_HCWA = Medicare Helping Children with Autism; (d) IS = Independent Schools; (e) MBS_BS = Medicare Better Start; (f) MBS_AHS = Medicare Allied Health for Aboriginal and Torres Strait Islander; (g) TP = Third Party.*

Participants, however, varied in their familiarity with PFMs, with 6.6 % of participants familiar with all seven PFMs presented in the survey and 4.1 % familiar with only one PFM. Familiarity was also associated with career experience (see Supplementary Material VI: Years of experience [career] and familiarity of PFM [accumulative number of PFMs known] reported by participants), with those participants practising over longer timeframes (i.e., >6 years) being familiar with more PFMs compared to early career participants (i.e. 0–5 years) who reported being familiar with up to two PFMs (82.4 %). Three PFMs were familiar to at least 60 % of participants (see Supplementary Material V: Participant responses for public funding models reported as “*familiar*”) and, therefore, progressed to mixed effects logistics regression. Four contemporary PFMs, therefore, were removed from our analyses as they did not meet our criterion value set at greater than 40 % unfamiliar (unf): (a) MBS_BS, unf = 47.1 %; (b) MBS_AHS, unf = 63.6 %; (c) TP, unf = 80.2 %; and (d) IS, unf = 47.1 % (see Fig. 1).

Additionally, the three PFMs identified as the three top-ranking PFMs in achieving SLP goals (i.e., ranked in order according to percentage of times they were identified by participants in the top three) had low percentages of unfamiliarity (i.e., these PFMs were familiar to participants), for example: (a) NDIS, unf = 0 %; (b) MBS_CDMP, unf = 7.4 %; & (c) MBS_HCWA, unf = 29.8 % (see Fig. 1). The three top-ranking PFMs in achieving SLP goals (see Fig. 1) were heavily influenced by familiarity of PFMs.

### 3.3 Odds ratios to identify incongruent public funding model schemes

Our analyses used odds ratios with 95 % confidence intervals for pairwise comparison of PFMs (see Table 3).

**Table 3:** Odds ratios with 95% confidence intervals comparing familiar PFMs from a mixed effects logistics regression of participants responding “does not align” (major comparisons of interest shown).

PFM Scheme^a,b,c^	Baseline^a,b,c^	Estimate	Odds Ratio 95 % CI	p-value
MBS_CDMP	BL1 = NDIS	4.92	2.94, 8.26	<0.001
NDIS	BL3 = MBS_HCWA	1.50	0.88, 2.37	1.31
MBS_CDMP	BL3 = MBS_HCWA	7.40	4.34, 12.64	<0.001

Note: CI = confidence interval; For complete dataset of odd ratios, see Supplementary Material VII: Complete Odds Ratio with 95 % CI comparing familiar PFMs from a mixed effects logistics regression of participants responding “does not align”; ^a^ NDIS = National Disability Insurance Scheme; ^b^ MBS_CDMP = Medicare Benefit Schedule – Chronic Disease Management Plan; ^c^ MBS_HCWA = Medicare Benefit Schedule - Helping with Children with Autism.

In comparing NDIS (as baseline) with MBS_CDMP, the odds ratio was 4.92 (95 % CI 2.94, 8.26); the odds of incongruence with scientific evidence (i.e., not aligning with evidence-based practice) were 4.92 times higher for MBS_CDMP than NDIS. In analysing our third baseline (e.g., BL_3_ = MBS_HCWA), the odds ratio between NDIS and MBS_HCWA identified that both PFMs aligned with scientific evidence with estimates of 1.50 (95 % CI 0.88, 2.37); additionally, mixed effects logistics regression analysis showed no significant difference between the two PFMs. Conversely, MBS_CDMP was 7.40 (95 % CI 4.34, 12.64) times higher in not aligning with best available scientific evidence when compared to MBS_HCWA.

## 4 Discussion

This study aimed to determine whether contemporary PFMs accessed by families for children and young persons through Australian private-practice SLP providers were congruent with contemporary scientific evidence for SLP management. From the seven contemporary PFMs investigated, only three PFMs were familiar to 60 % of participants. Our research suggests that participants with <6 years of career experience have little or no awareness of alternative contemporary PFMs; this provides educational opportunities for pre-service SLP training and Certified Practising Speech Pathologists with less than six years’ experience. Apart from identifying which current PFMs were either familiar or unfamiliar to Certified Practising Speech Pathologists, our statistical analysis also revealed greater incongruence (i.e., misalignment) with scientific evidence for MBS_CDMP than for NDIS and MBS_HCWA. Additionally, MBS_CDMP was the only PFM where all SLP treatment areas did not align with the recommended differential treatment intensity evidence (see Table 1), a position supported by Law and Conti-Ramsden [8] where both researchers advocated that six hours of therapy was not enough for effective SLP management.

As our research illustrates, current PFM legislation and guidelines allow access by children and young persons with communication and swallowing disorders to PFMs as outlined in Table 1; however, anecdotal and scientific evidence suggests their eligibility and access to PFMs is ambiguous and open to interpretation by funding gatekeepers (i.e., General Practitioners, National Disability Insurance Agency planners, Local Area Coordinators and policy architects who design PFMs; [2]). Funding gatekeepers, however, may determine eligibility to funding criteria without knowledge of the evidence base for SLP practice areas [2]. Our mapping highlighted that: (a) all three familiar PFMs allow provision of access across all SLP clinical practice areas, however this may be dependent on complexity, chronicity and/or functional impact of diagnosis to meet PFM eligibility (e.g., autism spectrum disorder, childhood apraxia of speech); (b) NDIS aligned with the scientific evidence for SLP management for all (100 %) identified studies and clinical practice areas; (c) eight out of fourteen studies and clinical practice areas (57 %) aligned with MBS_HCWA; and (d) no studies or clinical practice areas (0 %) aligned with the scientific evidence for SLP management under MBS_CDMP. Our previous research [2], [33] together with results from the survey reported here conclusively affirms incongruence between MBS_CDMP and the myriad of scientific evidence for effective SLP management.

Since the introduction of MBS_CDMP for speech-language pathologists in 2004, there has been a significant increase in demand for the relevant Medicare Benefit Schedule item. Between July 2004 and July 2023, a total of 1,906,656 SLP services (93 % for children) were reported as being delivered using this government subsidised rebate, at a total cost of AUD $109.1 million to taxpayers [50]. Given this significant investment, we therefore offer the following recommendations to maximise: (a) health outcomes within the shortest possible timeframe; and (b) return on investment for taxpayers.

### 4.1 Recommendation 1: Review to align public funding models with scientific evidence

Given our findings of incongruence with best-available scientific evidence, we propose that: (a) review of MBS_CDMP be prioritised to align SLP management with scientific evidence; and (b) MBS_CDMP allow for intensification of service delivery in using contemporary PFMs to align with evidence-based practice; this could also extend to other contemporary and future designed PFMs. Currently MBS_CDMP’s number of rebated sessions is restricted to only five sessions per calendar year. We argue that the design of this PFM does not align with best-available scientific evidence and fails to: (a) achieve optimal healthcare outcomes recommended by the evidence; and (b) maximise returns to the Australian taxpayer in the shortest possible timeframe. The literature supports timely, early intervention [51], [52]; a goal which could be achieved through intensifying service delivery. Given the potential chronicity and pervasiveness of communication and swallowing disorders for children and young persons and its direct impact on academic, social and employment outcomes [53], [54], we propose increasing the current practice of accessing MBS_CDMP (and other similar PFMs) over long time frames, to a shorter more intensive approach. For example, under current MBS_CDMP legislation, children and young persons with chronic communication and swallowing disorders may access SLP services over many years (i.e., four years or longer). In calculating the absolute number over four years of access to MBS_CDMP (i.e., 20 sessions in total over four years), we propose delivering the same amount of SLP services in shorter timeframes (i.e., intensive intervention) to align with best scientific evidence (see Table 1). A precedent already exists with lifetime limits of up to 20 sessions available for access to allied healthcare services for this population (e.g., Complex Neurodevelopmental Disorders and Eligible Disabilities, formerly incorporating Medicare’s Helping Children with Autism and Better Start for Children with Disability [37]). We propose that MBS_CDMP reflect this precedent and align with the number of sessions required to meet best available scientific evidence of up to 20 sessions in total (see Table 1). This will not only meet government health-economic objectives of equity, efficiency and acceptability, but will ensure children and young persons can maximise future academic, social and employment opportunities. Finally, access to PFMs should apply equally across all communication and swallowing disorders, which may not currently be the case as already reported in the literature [2]. By failing to act in a timely and efficient manner, taxpayers are burdened with prolonged economic, academic, social and employment costs [10], [55].

### 4.2 Recommendation 2: Include other prevalent communication and swallowing disorders in criteria for access to public funding models

Regardless of socio-economic status, communication and swallowing are basic human rights [56]. Many communication and swallowing disorders, however, are misunderstood by funding gatekeepers [2], [33], [57], [58], which can mean that children, young persons and their families fail to access critical healthcare in a timely manner through PFMs [2]. Many communication and swallowing disorders align with PFM criteria due to their chronicity and complexity (e.g., specific learning difficulties [i.e., developmental language disorder], dyslexia and dysgraphia; speech sound disorders [i.e., articulation disorder, phonological disorders andchildhood apraxia of speech]; fluency disorders of speech [i.e., stuttering]; and swallowing/feeding disorders [i.e., paediatric feeding disorder]; [33]; see Table 1). Therefore, advocating to funding gatekeepers and policy makers for future inclusion of communication and swallowing disorders should be a priority for stakeholders (e.g., Speech Pathology Australia, consumer groups, academics, service providers).

### 4.3 Recommendation 3: Education opportunities

Even with the diversity of public funding typologies available to children and young persons with communication and swallowing disorders [2], our research identified knowledge gaps in contemporary PFMs by Certified Practising Speech Pathologists. Therefore, an opportunity exists to provide professional education via tertiary training during pre-service SLP programs so that graduates are fully versed with the array of funding typologies (including PFM criteria) available to individuals with communication and swallowing disorders and not just the top three PFMs identified by this study (see Fig. 1 and Table 3).

Our research has raised the need to ensure that both practice and policy address equitable access to public funding for all children and young persons with communication and swallowing needs. This study, therefore, invites future research and policy development in advancing principles of equity, efficiency and acceptability [2] to align congruence between contemporary PFMs with scientific evidence of best practice SLP management. Measures to progress such stratagems include: (a) collaborations/education opportunities (e.g., webinars, workshops, symposiums) aimed to support funding gatekeepers and their knowledge of SLP management and suitable PFMs; and (b) dialogue and collaborations with policy architects to improve current PFM criteria and future PFM design.

Our study is not without limitations. The sample size (i.e., n = 121) did not meet our proposed sample size for adequate power; however, the effects in the study were greater than anticipated in sample size planning and differences between PFMs were detected. A second limitation is the rapidly changing policy environment. Since the inception of this study, the SLP profession has successfully advocated for additional items legislated under Medicare Benefit Schedule (e.g., Allied Health Telehealth and Phone Services; Multidisciplinary Case Conferencing; Rhinology – see Supplementary Material III: Part B, Contemporary public funding models; [37]) for access by children and young persons through Australian private-practice SLP providers. These PFMs have not been included in this study. As of 1st March 2023, two PFMs that were investigated in our study (i.e., Helping Children with Autism and Better Start for Children with Disability) have been re-categorised under a new Medicare Benefit Scheme item titled Complex Neurodevelopmental Disorders and Eligible Disabilities [32].

## 5 Conclusion

This study is the first to report on the (in)congruence between contemporary PFMs for access to Australian private SLP services by children and young persons and the evidence base for effective SLP management. This novel research indicated that: (a) speech-language pathologists’ familiarity of PFMs vary; and (b) PFMs differ in their ability to meet rigorous scientific evidence for SLP management. Ensuring PFMs support an evidence-based pattern of practice will ensure that Australian government objectives of health-economic principles of equity, efficiency and acceptability are realised. In particular, PFMs should offer more intensive configurations of service delivery, specifically additional services delivered within each year to align with best available scientific evidence-based practice.

## Disclosure statement

The authors report no conflict of interest.

## Funding

Tristan Nickless was funded by the Australian Government Research Training Program Scholarship.

## CRediT authorship contribution statement

**T. Nickless:** Conceptualization, Data curation, Writing – original draft, Writing – review & editing, Visualization, Investigation, Validation, Formal analysis, Methodology, Software. **B. Davidson:** Writing – review & editing, Supervision. **S. Finch:** Visualization, Writing – review & editing, Software. **L. Gold:** Writing – review & editing, Supervision. **R. Dowell:** Writing – review & editing, Supervision.

## Declaration of competing interest

The authors declare that they have no known competing financial interests or personal relationships that could have appeared to influence the work reported in this paper.
