# Supplementary material for: Aligned or misaligned: Are public funding models for speech-language pathology reflecting recommended evidence? An exploratory survey of Australian speech-language pathologists
Source: Health Policy Open. 2024 Mar 7;6:100117. doi: 10.1016/j.hpopen.2024.100117 (PMC10950885; doi:10.1016/j.hpopen.2024.100117)
Supplement: Supplementary data 1 [file mmc1.docx]

**SUPPLEMENTARY MATERIALS**

**Supplementary Material I: Survey**

Q1

**I will be answering the following questions with my knowledge of public funding in:**

- public practice (1)
- not-for-profit (NFP)/ non-governmental organisation (NGO) (2)
- private-practice practice (3)
- combination of settings (4)

Q2

**I will be answering the following questions with my knowledge of public funding within Australian private-practice practice for:**

- children & adolescents (1)
- adults (2)

Q3

**I am:**

- a financial member of Speech Pathology Australia and a Certified Practising Speech Pathologist (CPSP) (1)
- a financial member of Speech Pathology Australia however NOT a Certified Practising Speech Pathologist (CPSP) (2)
- not a member of Speech Pathology Australia (3)

Q4

**I currently practice the MAJORITY of my time in postcode (i.e. registered office/clinic; if mobile service or part time, postcode you spend majority of time):**

- Postcode (1) ________________________________________________

Q5

**To which gender identity do you most identify?**

- Male (1)
- Female (2)
- Non-binary (3)
- Prefer not to say (4)

Q6

**What is your age?**

- 20-29 years (1)
- 30-39 years (2)
- 40-49 years (3)
- 50-59 years (4)
- 60-69 years (5)
- ≥ 70 years (6)

Q7

**Which job/position title reflects your current role?**

- Clinician (1)
- Senior Clinician (2)
- Manager & Clinician (3)
- Manager (no clinical role) (4)
- Owner of Practice & Clinician (5)
- Owner of Practice (no clinical role) (6)

Q8

**How many years of clinical experience have you had as a speech pathologist (entire career)?**

- 0-5 (1)
- 6-10 (2)
- 11-15 (3)
- 16-20 (4)
- 21-30 (5)
- 31+ (6)

Q9

**How many years of experience have you had in your main clinical role (current)?**

- 0-5 (1)
- 6-10 (2)
- 11-15 (3)
- 16-20 (4)
- 21+ (5)

Q10

**What hourly rate does your practice charge for a standard consultation to access speech pathology services ($AUD)?**

- < $100 (1)
- $101-150 (2)
- $151-200 (3)
- $200+ (4)

Q11

**In thinking about your OWN caseload, estimate (as a percentage) how much public funding can be attributed to total SLP revenue per annum?**

- 0 (1)
- 1-10 (2)
- 11-20 (3)
- 21-30 (4)
- 31-40 (5)
- 41-50 (6)
- 51-60 (7)
- 61-70 (8)
- 71-80 (9)
- 81-90 (10)
- 91+ (11)
- Not enough experience to estimate (12)

Q12

**Other than clinical need, access to public funding can sometimes depend on the family's characteristics. How much do you agree with the following statements? Successful access to funding is reliant on:**

|  |  |
| --- | --- |
| i) the advocacy skills of family members (1) | ▼ Strongly agree (6) ... Strongly disagree (10) |
| ii) family members understanding and using the right language (2) | ▼ Strongly agree (6) ... Strongly disagree (10) |
| iii) the education level of family members (3) | ▼ Strongly agree (6) ... Strongly disagree (10) |
| iv) a family's knowledge of a funding system (4) | ▼ Strongly agree (6) ... Strongly disagree (10) |
| v) the knowledge of communication and swallowing disorders by funding decision makers (5) | ▼ Strongly agree (6) ... Strongly disagree (10) |

Q13 **Are there other factors that determine access to funding (other than clinical need), not listed here?**

________________________________________________________________

________________________________________________________________

________________________________________________________________

________________________________________________________________

________________________________________________________________

Q14

**Please rank in order from 1 to 4, which factors consume the most of your time in accessing public funding provisions for your clients (where '1' has most impact and '4' being least impact. On your device, move the tiles into your rank order.)**

______ Time spent explaining public funding model criteria to client (1)

______ Time spent liaising with decision makers of funding (2)

______ Time spent completing administration requirements for public funding models (3)

______ Time spent navigating public funding model criteria (4)

Q15

**Please rate each of the following public funding models according to whether or not each model's funding provisions align with best available scientific evidence for SLP assessment & intervention.**

| National Disability Insurance Scheme (1) | ▼ Does not align (1) ... Not familiar with funding model (4) |
| --- | --- |
| Medicare - Chronic Disease Management Plan (2) | ▼ Does not align (1) ... Not familiar with funding model (4) |
| Medicare - Helping Children with Autism (3) | ▼ Does not align (1) ... Not familiar with funding model (4) |
| Medicare - Better Start (4) | ▼ Does not align (1) ... Not familiar with funding model (4) |
| Medicare - Allied Health Services for Aboriginal and Torres Strait Islander Decent (5) | ▼ Does not align (1) ... Not familiar with funding model (4) |
| Independent School Funding (6) | ▼ Does not align (1) ... Not familiar with funding model (4) |
| Third Party Funding (i.e. Motor Accident Commissions, Dept. of Veteran Affairs, Workcover) (7) | ▼ Does not align (1) ... Not familiar with funding model (4) |

Q16

**Imagine you would like to improve *TRUST* of speech-language pathologists amongst other stakeholders. How would you achieve this? Rank from 1 to 4 in order of importance, where 1 is 'most important' and 4 is 'least important'.**

______ Advocate that funding decision makers listen to experts i.e. peak bodies such as Speech Pathology Australia, clinical experts, academics (1)

______ Develop trust with clients by including them in public funding decision making (2)

______ Make autonomous decisions as an evidence based healthcare professional (3)

______ Improve communication(s) about SLP service provisions (4)

Q17

**Please rank in order from 1 to 7 how the following consequences of funding models impact your clients' funding provisions (where '1' has most impact and '7' being least impact).**

______ Consistency of funding arrangements by decision makers (1)

______ Improved interdisciplinary communication between professionals (2)

______ Funding decision makers knowing speech-language pathologists' scope of practice (3)

______ Consultation between funding decision makers and speech-language pathologists in setting speech pathology goals (4)

______ Administrative demands on speech-language pathologists of funding model reporting requirements (5)

______ Navigation of funding model criteria (6)

______ Demand for SLP services and impact on workforce (7)

Q18

**Please rank your preference of public funding model in achieving speech pathology client goals for *ONLY THREE* (3) out of the seven (7) funding models (where '1' is your most preferred and '3' is your least preferred). NOTE: Drag only ONE (1) response per Ranking Box.**

| Rank 1 | Rank 2 | Rank 3 |
| --- | --- | --- |
| ______ National Disability Insurance Scheme (1) | ______ National Disability Insurance Scheme (1) | ______ National Disability Insurance Scheme (1) |
| ______ Medicare - Chronic Disease Management Plan (2) | ______ Medicare - Chronic Disease Management Plan (2) | ______ Medicare - Chronic Disease Management Plan (2) |
| ______ Medicare - Helping Children with Autism (3) | ______ Medicare - Helping Children with Autism (3) | ______ Medicare - Helping Children with Autism (3) |
| ______ Medicare - Better Start (4) | ______ Medicare - Better Start (4) | ______ Medicare - Better Start (4) |
| ______ Medicare - Allied Health Services for Aboriginal and Torres Strait Islander Decent (5) | ______ Medicare - Allied Health Services for Aboriginal and Torres Strait Islander Decent (5) | ______ Medicare - Allied Health Services for Aboriginal and Torres Strait Islander Decent (5) |
| ______ Independent School Funding (6) | ______ Independent School Funding (6) | ______ Independent School Funding (6) |
| ______ Third Party Funding (i.e. Motor Accident Commissions, Dept. of Veteran Affairs, Workcover) (7) | ______ Third Party Funding (i.e. Motor Accident Commissions, Dept. of Veteran Affairs, Workcover) (7) | ______ Third Party Funding (i.e. Motor Accident Commissions, Dept. of Veteran Affairs, Workcover) (7) |

Q19

**In the future, would you be prepared to provide deidentified data relating to your clients' funding to a third party organisation to allow comparisons and analysis for advocacy?**

- No (18)
- Yes (19)

Q20

**To what extent has Covid 19 positively or negatively impacted on your client's ability to access public funding provisions?**

- Extremely positive (1)
- Somewhat positive (2)
- Neither positive nor negative (3)
- Somewhat negative (4)
- Extremely negative (5)

Q21

**To what extent has Covid 19 increased or decreased the amount of public funding allocated to clients?**

- Significantly increased (1)
- Somewhat increased (2)
- Neither increased nor decreased (3)
- Somewhat decreased (4)
- Significantly decreased (5)

Q22

**Should you wish to receive a summary of results and/or a copy of future publications, please provide your email in the textbox below.**

________________________________________________________________

Q23

**Thank you for your participation. It is greatly appreciated. If you would like to leave any further comments or remarks, please feel free to leave them here.**

________________________________________________________________

________________________________________________________________

________________________________________________________________

________________________________________________________________

________________________________________________________________
