# Supplementary material for: Aligned or misaligned: Are public funding models for speech-language pathology reflecting recommended evidence? An exploratory survey of Australian speech-language pathologists
Source: Health Policy Open. 2024 Mar 7;6:100117. doi: 10.1016/j.hpopen.2024.100117 (PMC10950885; doi:10.1016/j.hpopen.2024.100117)
Supplement: Supplementary data 2 [file mmc2.docx]

**Supplementary Material II: Specific survey questions**

| Survey Question | Response options |
| --- | --- |
| Please rate each of the following public funding models according to whether or not each model’s funding provisions align with best available scientific evidence for SLP assessment & intervention. ^a^ | 1. does not align; 2. aligns; 3. neither aligns nor does not align; or 4. not familiar |
| Please rank your preference of public funding model in achieving speech pathology client goals for ONLY THREE (3) out of the seven (7) funding models. ^b^ | (1) ‘1’ is most preferred; and  (3) ‘3’ is least preferred |

*Note: ^a^ see Supplementary Material I: Survey, question 15; ^b^ see Supplementary Material I: Survey, question 18.*
