# Supplementary material for: Aligned or misaligned: Are public funding models for speech-language pathology reflecting recommended evidence? An exploratory survey of Australian speech-language pathologists
Source: Health Policy Open. 2024 Mar 7;6:100117. doi: 10.1016/j.hpopen.2024.100117 (PMC10950885; doi:10.1016/j.hpopen.2024.100117)
Supplement: Supplementary data 3 [file mmc3.docx]

**Supplementary Material III: Contemporary public funding models**

**Part A**

This study investigated the following contemporary PFMs available to children and young persons with communication and swallowing for access to Australian private-practice services at the time of research. The below information details each PFM used in our research:

**Public funding models investigated in study**

| Study Reference | Public Funding Name | PFM Criteria |
| --- | --- | --- |
| NDIS | National Disability Insurance Scheme ^a^ | - *Prospective Participants* must meet the following criteria to be eligible for participation and funding under the NDIS ^a^:   - access criteria (section 21)   - age requirements (section 22)   - residency requirements (section 23) - disability requirements (section 24) |
| MBS_CDMP | Medicare Benefit Scheme - Chronic Disease Management Plan ^b^ | - MBS item number: 10970 ^b^   - condition is (a) chronic and (b) complex   - referred by GP under GP management plan and Team Care Arrangement   - up to five services per calendar year   - service is in-person   - individual sessions   - person is not an admitted patient of a hospital   - service is at least 20 minutes in duration   - eligible speech pathologist provides a written report to referring medical practitioner: (a) if the service is the only service under the referral; (b) the first or the last service under the referral - not to be used in conjunction with private health insurance benefit |
| MBS_HCWA | Medicare Benefit Scheme - Helping Children with Autism ^b, c^ | - MBS item number: 82005 (assessment) ^b^   - service is in-person   - individual sessions   - person is not an admitted patient of a hospital   - service is at least 50 minutes in duration   - assessment: up to 4 services in total prior to 13^th^ birthday - MBS item number 82020 (treatment) ^b^   - service is in-person   - individual sessions   - person is not an admitted patient of a hospital   - service is at least 30 minutes in duration - treatment: up to 20 services in total prior to 15^th^ birthday |
| IS | Independent Schools ^d, e^ | - Independent schools are eligible for specific funding from Commonwealth, and state and territory governments to support the education of students with disability. ^e^ - The current Australian Government school funding model provides a loading for students with disability. It is determined using the [Nationally Consistent Collection of Data on School Students with Disability (NCCD)](https://www.nccd.edu.au/) data. The NCCD identifies students eligible to receive an adjustment for their disability. Adjustments are based on appraisal by teachers with supporting evidence to support the level of particular adjustment. e   There are four levels of adjustment, with (b) to (c) funded under the Commonwealth disability loading e:   1. Support provided within a Quality Differentiated Teaching Practice – support through active monitoring and adjustments are not greater than those used to meet the needs of diverse learners 2. Supplementary – adjustments that are supplementary to the strategies and resources already available for all students within the school 3. Substantive – essential adjustments and considerable adult assistance  - Extensive – students with disability and very high support needs |
| MBS_BS | Medicare Benefit Scheme - Better Start ^b^ | - MBS item number: 82005 (assessment) ^b^   - service is in-person   - individual sessions   - person is not an admitted patient of a hospital   - service is at least 50 minutes in duration   - assessment: up to 4 services in total prior to 13^th^ birthday - MBS item number: 82020 (treatment) ^b^   - service is in-person   - individual sessions   - person is not an admitted patient of a hospital   - service is at least 30 minutes in duration - treatment: up to 20 services in total prior to 15^th^ birthday |
| MBS_AHS | Medicare Benefit Scheme - Allied Health Services for Aboriginal Torres Strait Islander Decent with Health Checks ^b^ | - MBS item number: 81360 ^b^   - Medical practitioner has undertaken a health assessment and has identified need for follow up allied health service; or shared care plan identified need for follow up allied health service   - referred by medical practitioner using referral form (or form that substantially complies) issued by Department of Health   - up to five services per calendar year   - service is in-person   - individual sessions   - person is not an admitted patient of a hospital   - service is at least 20 minutes in duration - eligible speech pathologist provides a written report to referring medical practitioner: (a) if the service is the only service under the referral; (b) the first or the last service under the referral |
| TP | Third Party ^f^ | Examples vary and may include funding through criteria set by Department of Veteran Affairs and state based transport accident commissions. |

Notes: ^a^ (National Disability Insurance Scheme Act [Cth], 2013); ^b^ MBS = Medicare Benefit Schedule (Department of Health and Aged Care, 2023); ^c^ As at March 2023, this Medicare Benefit Schedule item has now been superseded by Complex Neurodevelopmental Disorder & Eligible Disabilities; ^d^ such as Independent Schools Victoria, Students with Disabilities (see Nickless et al., 2023a, Table I); ^e^ Independent Schools Australia, 2023; ^f^ such as such as Transport Accident Commission Victoria (see Nickless et al., 2023a, Table I).

**Part B**

The following PFMs have subsequently become available to children and young persons with communication and swallowing for access to Australian private-practice-practice services post survey:

**Newly introduced PFMs since completion of study**

| Public Funding Name | PFM Criteria |
| --- | --- |
| Medicare Benefits Scheme - Allied Health Telehealth and Phone Service ^a^ | - MBS item number: 93000 & 93013 ^a^   - condition is (a) chronic and (b) complex   - referred by GP under GP management plan and Team Care Arrangement   - up to five services per calendar year   - service is via: (a) telehealth (MBS:93000); or (b) phone (MBS:93013)   - individual sessions   - person is not an admitted patient of a hospital   - service is at least 20 minutes in duration - eligible speech pathologist provides a written report to referring medical practitioner: (a) if the service is the only service under the referral; (b) the first or the last service under the referral |
| Medicare Benefit Scheme - Allied Health Services for Aboriginal Torres Strait Islander Decent with Health Checks ^a^ | - MBS item number: 93048 & 93061 ^a^   - Medical practitioner has undertaken a health assessment and has identified need for follow up allied health service; or shared care plan identified need for follow up allied health service   - referred by medical practitioner using referral form (or form that substantially complies) issued by Department of Health   - up to five services per calendar year   - service is via: (a) telehealth (MBS:93048); or (b) phone (MBS: 93061)   - individual sessions   - person is not an admitted patient of a hospital   - service is at least 20 minutes in duration - eligible speech pathologist provides a written report to referring medical practitioner: (a) if the service is the only service under the referral; (b) the first or the last service under the referral |
| Medicare Benefits Scheme - Complex Neurodevelopmental Disorder & Eligible Disabilities ^a, b^ | - MBS item number: 82005 (assessment) ^a^   - service is in-person   - individual sessions   - person is not an admitted patient of a hospital   - service is at least 50 minutes in duration   - assessment: up to 8 services in total prior to 25^th^ birthday - MBS item number 82020 (treatment) ^a^   - service is in-person   - individual sessions   - person is not an admitted patient of a hospital   - service is at least 30 minutes in duration   - treatment: up to 20 services in total prior to 25^th^ birthday |
| Medicare Benefits Scheme - Service for Assessment and Treatment of Complex Neurodevelopmental Disorder & Eligible Disabilities ^a^ | - MBS item number: 93033 (assessment) ^a^   - service is via telehealth   - individual sessions   - person is not an admitted patient of a hospital   - service is at least 50 minutes in duration   - assessment: up to 8 services in total prior to 25^th^ birthday - MBS item number: 93036 (treatment) ^a^   - service is via telehealth   - individual sessions   - person is not an admitted patient of a hospital   - service is at least 30 minutes in duration   - treatment: up to 20 services in total prior to 25^th^ birthday |
| Medicare Benefits Scheme - Service for Assessment and Treatment of Complex Neurodevelopmental Disorder & Eligible Disabilities ^a^ | - MBS item number: 93041 (assessment) ^a^   - service is via phone   - individual sessions   - person is not an admitted patient of a hospital   - service is at least 50 minutes in duration   - assessment: up to 8 services in total prior to 25^th^ birthday - MBS item number:93044 (treatment) ^a^   - service is via phone   - individual sessions   - person is not an admitted patient of a hospital   - service is at least 30 minutes in duration   - treatment: up to 20 services in total prior to 25^th^ birthday |
| Medicare Benefits Scheme - Rhinology ^a^ | - MBS item number: 41764 ^a^   - Written request made by a specialist otolaryngologist to assist in the diagnosis, treatment and management of laryngeal or related disorders   - Administration of nasendoscopy or sinoscopy or fiberoptic examination of the nasopharynx or larynx, one or more of these procedures, unilateral or bilateral examination   - service is performed in a medical facility   - service is in-person   - service is performed individually - eligible speech pathologist provides (a) recorded dynamic images of, and a copy of the results of, the service; and (b) relevant written comments about the results |
| Medicare Benefit Scheme – Individual Allied Health Services for Chronic Disease Management: Case Conferences ^a^ | - MBS item number: 10955 ^a^   - attendance as a multidisciplinary case conference team   - at least 15 minutes, but less than 20 minutes   - in-person, via telehealth or via phone - MBS item number: 10957 ^a^   - attendance as a multidisciplinary case conference team   - at least 20 minutes, but less than 40 minutes   - in-person, via telehealth or via phone - MBS item number: 10959 ^a^   - attendance as a multidisciplinary case conference team   - at least 40 minutes   - in-person, via telehealth or via phone |
| Medicare Benefit Scheme – Complex Neurodevelopmental Disorder and  Disability Services: Allied Health: Case Conferences ^a^ | - MBS item number: 82001^a^   - attendance as a multidisciplinary case conference team   - at least 15 minutes, but less than 20 minutes   - in-person, via telehealth or via phone - MBS item number: 82002 ^a^   - attendance as a multidisciplinary case conference team   - at least 20 minutes, but less than 40 minutes   - in-person, via telehealth or via phone - MBS item number: 82003 ^a^   - attendance as a multidisciplinary case conference team   - at least 40 minutes   - in-person, via telehealth or via phone |

*Notes: ^a^ Department of Health and Aged Care, 2023; ^b^ As of 1 March 2023, both MBS_HCWA and MBS_BS have been categorised under a new Medicare Benefits Schedule (MBS) item titled Complex Neurodevelopmental Disorders* (Department of Health and Aged Care, 2023)*.*

References:

Department of Health and Aged Care. (2023, April 5). *Medicare Benefit Schedule*. https://www9.health.gov.au/mbs/search.cfm?q=10970&sopt=I

Independent Schools Australia. (2023). *Independent Schools Australia*. Students with a Disability. https://isa.edu.au/our-sector/diversity/students-with-disability/

National Disability Insurance Scheme Act (Cth) (2013). Compilation No. 8, October 2016 https://www.legislation.gov.au/Details/C2016C00934

Nickless, T., Gold, L., Dowell, R., & Davidson, B. (2023). Public purse, private service: The perceptions of public funding models of Australian independent speech-language pathologists. *International Journal of Speech-Language Pathology*, *25*(3), 462–478. https://doi.org/10.1080/17549507.2023.2213864
