# Supplementary material for: Aligned or misaligned: Are public funding models for speech-language pathology reflecting recommended evidence? An exploratory survey of Australian speech-language pathologists
Source: Health Policy Open. 2024 Mar 7;6:100117. doi: 10.1016/j.hpopen.2024.100117 (PMC10950885; doi:10.1016/j.hpopen.2024.100117)
Supplement: Supplementary data 4 [file mmc4.docx]

**Supplementary IV: Participant characteristics**

| **Characteristic** | **Percent** |
| --- | --- |
| **Age (years)**  20-29  30-39  40-49  50-59  60-69 | 27.3  28.1  21.5  19.8  3.3 |
| **Career experience (years)**  0-5  6-10  11-15  16-20  21-30  30+ | 32.2  14.0  17.4  7.4  14.9  14.1 |
| **Position**  Manager (no clinical role)  Owner of practice (no clinical role)  Manager & clinician  Senior clinician  Clinician  Owner of practice & clinician | 0.8  2.5  4.1  11.6  38.8  42.2 |
| **Current position (years)**  0-5  6-10  11-15  16-20  21+ | 56.2  10.7  10.7  7.5  14.9 |
| **Geographic location ^a^**  Major city  Regional  Remote | 81.0  16.5  2.5 |

*Note: ^a^ classification of geographic remoteness using Australian Statistical Geographical Structure - Remoteness Structure (Australian Institute of Health and Welfare, 2004).*

Reference:

Australian Institute of Health and Welfare. (2004). *Rural, regional and remote health: A guide to remoteness classifications* (AIHW cat. no. PHE 53). AIHW.
