# Supplementary material for: Aligned or misaligned: Are public funding models for speech-language pathology reflecting recommended evidence? An exploratory survey of Australian speech-language pathologists
Source: Health Policy Open. 2024 Mar 7;6:100117. doi: 10.1016/j.hpopen.2024.100117 (PMC10950885; doi:10.1016/j.hpopen.2024.100117)
Supplement: Supplementary data 5 [file mmc5.docx]

**Supplementary Material V: Participant responses for public funding models reported as “*familiar*”**

| **Participant response** | **Percent ^a^** |
| --- | --- |
| **Familiarity of PFM Scheme**  NDIS ^b^  MBS_CDMP ^c^  MBS_HCWA ^d^  MBS_BS ^e^  MBS_AHS ^f^  IS ^g^  TP ^h^ | 100.0  92.6  70.2  52.9  36.4  52.9  19.8 |

*Notes: ^a^ For the purpose of decimal rounding, cumulative percentages of PFMs may not equal 100; ^b^ NDIS = National Disability Insurance Scheme; ^c^ MBS_CDMP = Medicare Benefit Schedule – Chronic Disease Management Plan; ^d^ MBS_HCWA = Medicare Benefit Schedule – Helping Children with Autism; ^e^ MBS_BS = Medicare Benefit Schedule – Better Start; ^f^ MBS_AHS = Medicare Benefit Schedule – Allied Health Services for Aboriginal Torres Strait Islander Decent with Health Checks; ^g^ IS = Independent Schools; ^h^ TP = Third Party.*
