# Supplementary material for: Aligned or misaligned: Are public funding models for speech-language pathology reflecting recommended evidence? An exploratory survey of Australian speech-language pathologists
Source: Health Policy Open. 2024 Mar 7;6:100117. doi: 10.1016/j.hpopen.2024.100117 (PMC10950885; doi:10.1016/j.hpopen.2024.100117)
Supplement: Supplementary data 7 [file mmc7.docx]

**Supplementary Material VII: Complete Odds Ratio with 95% CI comparing familiar PFMs from a mixed effects logistics regression of participants responding “does not align”**


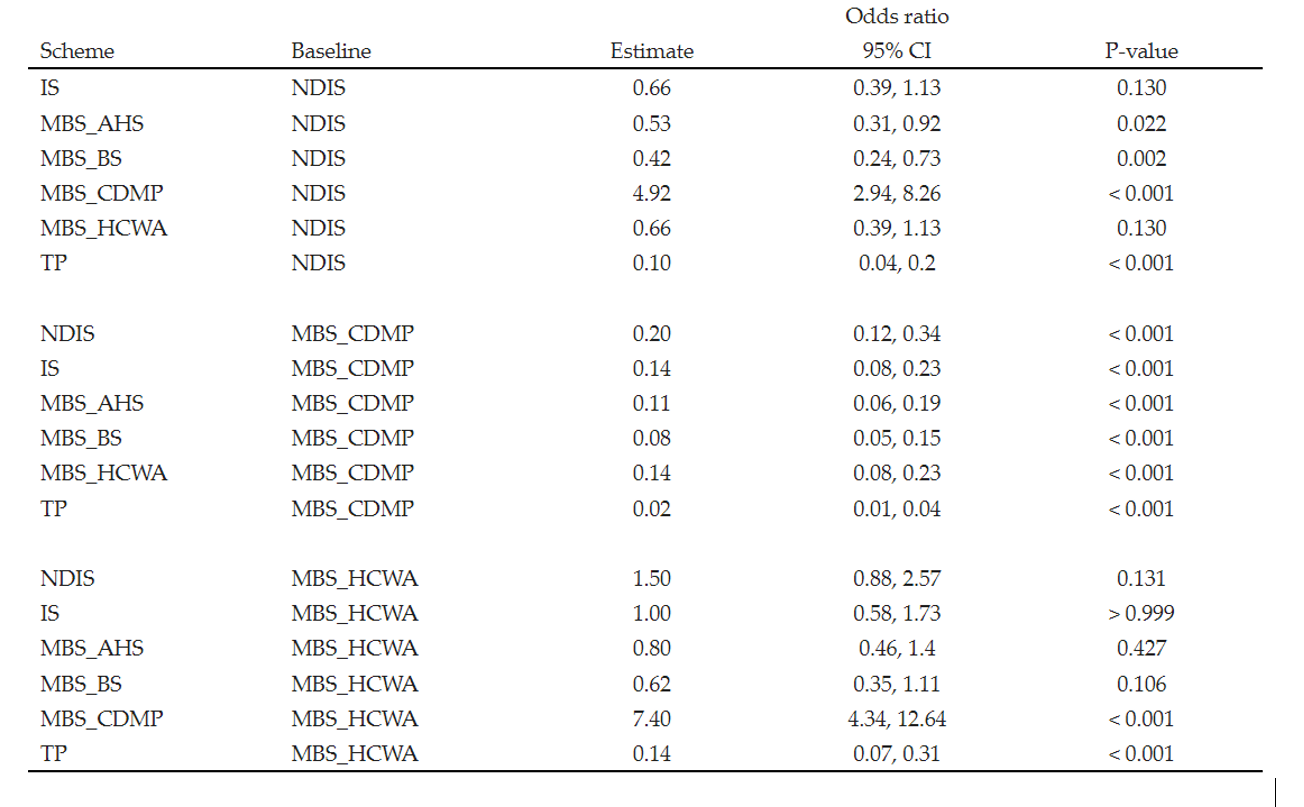


*Note: (a) NDIS = National Disability Insurance Scheme; (b) MBS_CDMP = Medicare Chronic Disease Management Plan; (c) MBS_HCWA = Medicare Helping Children with Autism; (d) IS=Independent Schools; (e) MBS_BS = Medicare Better Start; (f) MBS_AHS = Medicare Allied Health for Aboriginal and Torres Strait Islander; (g) TP = Third Party.*
